# Application of Treatment Response Biomarkers from Major Depression to Perinatal Depression

**DOI:** 10.3390/jpm15120607

**Published:** 2025-12-06

**Authors:** Wan Kwok, Melissa Wagner-Schuman, Tory Eisenlohr-Moul, Brandon Hage

**Affiliations:** 1 Department of Psychiatry, University of Illinois at Chicago, Chicago, IL 60612, USA; mwagner9@uic.edu (M.W.-S.); temo@uic.edu (T.E.-M.); hagebm@uic.edu (B.H.); 2 Department of Psychiatry, University of Cincinnati, Cincinnati, OH 45267, USA; 3 Department of Psychology, University of Illinois at Chicago, Chicago, IL 60612, USA

**Keywords:** perinatal depression, biomarker, major depressive disorder, postpartum depression

## Abstract

**Background/Objectives**: Perinatal depression poses significant risks to maternal and fetal health, yet biomarkers for treatment response in the field remain limited. Given the overlap in symptoms with major depressive disorder (MDD) and the comparatively more vast MDD literature, identifying promising MDD biomarkers for treatment response and examining corresponding perinatal depression biomarkers can reveal translational opportunities. **Methods**: PUBMED searches were conducted for individual biomarkers and MDD and perinatal depression, as well as with treatment response to antidepressant pharmacological treatment and neuromodulation treatments. When available, evidence from meta-analyses and systematic reviews were preferentially summarized. **Review**: This narrative review presents the current evidence on MDD and perinatal depression treatment response biomarkers, including brain-derived neurotrophic factor (BDNF), S100 calcium-binding protein B (S100B), electroencephalography, event-related potentials, metabolomics, hypothalamic–pituitary–adrenal axis hormones, neuroimaging markers, inflammatory markers, and neuroactive steroids. **Conclusions**: Biomarker research in MDD yields insights on promising biomarkers for treatment response, including BDNF, S100B, theta band density and cordance, inflammatory markers IL-8, CRP, and TNF- α, and neuroactive steroids.

## 1. Introduction

In recent years, perinatal and postpartum depression have garnered increasing recognition as an opportunity for clinical intervention, offering the potential to improve outcomes for both parent and infant. However, consensus regarding its etiology has yet to be reached. While some sources recognize peripartum depression as a distinct entity, possibly with different mechanistic causes than in primary mood disorders, others link it to unipolar or bipolar depression. Regardless of classification, depression during pregnancy and the postpartum period carries unique ramifications, including adverse maternal health outcomes [1,2] and risk of negative effects to fetal [3], infant [4], and child development [5,6].

While there is no perinatal or postpartum depression diagnosis in the DSM-5-TR, the specifier “with peripartum onset” is available for major depressive disorder. This is defined as the most recent depressive episode beginning during pregnancy or four weeks after delivery. Other more expansive definitions, and the widely accepted definition in the field of reproductive psychiatry, include any depressive episode beginning in pregnancy or within the first year after delivery. While most studies find the prevalence rates of perinatal/postpartum depression to be between 10 and 20%, there is evidence that these rates may be even higher, particularly in vulnerable patient populations [7]. Many patients interrupt or discontinue pharmacologic therapy during pregnancy and/or within the first two months postpartum [8], suggesting inadequacies in current treatment approaches. This gap between symptom burden and adequate treatment underscores the need for clinical research to guide clinical management and predict treatment outcomes in this population.

The need to identify biomarkers for psychiatric illnesses reflects growing recognition that identifying illness early may lead to both a better understanding of the causes of depression and improvements in treatment. Given the heterogeneity of depression, biomarkers that predict treatment response may be particularly useful. The literature on MDD biomarkers is comparatively more extensive than the literature specifically focused on perinatal depression. While perinatal depression has been conceptualized as a more homogeneous subtype of MDD [9,10,11], it remains a heterogeneous condition with overlapping mechanisms [12]. The perinatal period represents a distinct window in which early treatment may prevent negative short- and long-term outcomes for both the pregnant/postpartum patient and the infant [13,14]. By reviewing the literature on biomarkers for treatment response in MDD and perinatal depression, and by exploring translational opportunities, this review aims to identify biomarkers with the potential to enhance both diagnostic accuracy and treatment outcome.

## 2. Materials and Methods

This narrative review examines the current evidence of specific biomarkers that may predict treatment response for both unipolar depression not associated with pregnancy and perinatal depression. For the purpose of this review, biomarkers are defined as objective biological characteristics that can be measured with the goal of predicting the clinical outcome of an illness. Demographics and other variables related to treatment, such as prior episodes or antidepressant trials, are excluded. Studies focusing on bipolar depression were excluded to maintain diagnostic specificity and to reduce heterogeneity.

While symptomatically similar to unipolar depression, bipolar depression likely involves distinct neurobiological mechanisms and including such studies may confound the interpretation of findings.

A literature search was conducted using the PubMed database up to 30 September 2025. PubMed was chosen for its broad coverage of literature relevant to psychiatry, neuroscience, and perinatal research. Given the scope of this narrative review, PubMed was considered sufficient to identify the majority of relevant peer-reviewed studies. For each biomarker, PubMed searches were conducted with combinations of keywords for major depressive disorder and perinatal/postpartum depression association with treatment response, including antidepressant pharmacological treatments, ketamine/esketamine, and neuromodulation treatments—transcranial magnetic stimulation (TMS) and electroconvulsive therapy (ECT). Boolean operators were used for each combination of search terms (e.g., (“MDD” OR “major depressive disorder”) AND (“biomarker”) AND (“BDNF”) AND (“antidepressant” OR “TMS” OR “ketamine” OR “esketamine” OR “ECT”)). Additional relevant studies were identified through a manual review of reference lists. When available, evidence from meta-analyses and systematic reviews are preferentially summarized.

## 3. Review

### 3.1. Brain-Derived Neurotrophic Factor (BDNF)

#### 3.1.1. BDNF and MDD

BDNF is a protein that promotes the growth, differentiation, and survival of neurons. It plays a crucial role in synaptic plasticity. BDNF has been extensively studied as a potential biomarker for detecting MDD and treatment response in MDD. MDD has consistently been shown to be associated with lower peripheral BDNF levels compared to healthy individuals in several systematic reviews [15] and one meta-analysis showed strong evidence that BDNF levels are lower in depressed patients compared to healthy controls [16]. Typically, peripheral rather than central BDNF is studied for ease, with serum levels considered more reliable than plasma BDNF [17]. Given that a lower BDNF level has been reliably linked to MDD, investigation on its role in treatment response has been robust. There is also strong evidence supporting serum BDNF levels as possible predictors of MDD treatment response to antidepressant medications. In MDD patients, treatment response to antidepressants was reliably associated with a significantly increased BDNF serum level in comparison to treatment non-responders after treatment [15,17].

Evidence for BDNF modulation in response to neuromodulatory treatments is mixed. One meta-analysis that included studies involving a variety of treatments (medications, TMS, ECT, and vagal nerve stimulation (VNS)) demonstrated that BDNF blood levels increased significantly after treatment in patients with MDD [18]. Another meta-analysis focused on TMS and transcranial direct-current stimulation in treatment-resistant depression found no increase in change after treatment [19]. A randomized clinical trial compared plasma BDNF in patients with treatment-resistant depression receiving intravenous ketamine (*N* = 15) versus midazolam (*N* = 7) and found that ketamine responders had increased BDNF levels peaking at 240 min post-infusion compared to non-responders, while no association between BDNF was found in controls receiving midazolam [20]. In contrast, little association between BDNF and depressive symptom improvement was found in a meta-analysis examining treatment-resistant depression patients receiving a wide range of treatments, including ECT, rTMS, VNS, ketamine, lamotrigine, riluzole, and atypical antipsychotics [21]. Other findings suggest BDNF levels increase in depressed patients undergoing ECT, but this increase was not correlated with symptom improvement [22,23]. Current evidence suggests that BDNF may be useful in predicting both MDD and the treatment response to traditional antidepressant medications; however, in treatment-resistant depression and with neuromodulatory treatments, BDNF may not be a good predictor of treatment response.

#### 3.1.2. BDNF in Perinatal Depression

As one of the most commonly examined biomarkers for diagnosis and treatment response in MDD, peripheral BDNF has also been examined in perinatal depression. Similarly to MDD, studies have shown significantly decreased BDNF in patients with perinatal depression compared to controls [24]. However, only a few studies have examined treatment response. One study (*N* = 86) demonstrated that pregnant patients with perinatal depression treated with SSRIs had higher BDNF than those not treated with SSRIs [25]. However, among those treated with SSRIs, BDNF levels were not correlated with improvement in HAM-D scores.

Emerging research supports a link between BDNF levels and clinical improvement in perinatal depression. A recent novel RCT examined intravenous dexmedetomidine treatment for prevention of postpartum depression. It found that treatment significantly reduced the incidence of postpartum depression in participants receiving treatment (*N* = 169) compared to controls (*N* = 169). It also found significantly higher plasma BDNF levels in the postpartum period in participants receiving treatment compared to controls, suggesting that the antidepressant effect of this intervention is accompanied, and possibly mediated, by the increase in BDNF levels [26]. Given the substantial evidence supporting BDNF as a biomarker for treatment response in MDD for pharmacologic agents and the limited but positive evidence in perinatal depression, targeted research is needed to evaluate its translational utility in monitoring treatment response in perinatal depression more broadly.

### 3.2. S100 Calcium-Protein B (S100B)

#### 3.2.1. S100B in MDD

S100 calcium-binding protein B (S100B) is primarily produced by astrocytes. It has been studied as a biomarker related to neuroinflammation and decreased blood–brain barrier integrity. Extracellularly, the effects of S100B are concentration-dependent—at lower doses, it is thought to promote neurotrophic activity, and at high doses, contribute to neuroinflammation and cell injury. Elevated peripheral S100B levels have been demonstrated in a variety of psychiatric disorders [27]. Further, evidence suggests that elevated S100B levels are correlated with MDD severity [28]. It is thought that high levels of S100B may represent a compensatory mechanism for neuronal damage [29].

Many studies have demonstrated that, in patients diagnosed with MDD, higher S100B levels at baseline are associated with increased antidepressant response [30,31]. Additionally, higher S100B levels at baseline were associated with improved treatment response to imipramine and desvenlafaxine in melancholic depression [32]. Changes to S100B levels after treatment is more mixed. A meta-analysis did not find a consistent decrease in S100B post-treatment with antidepressants [28]. One study conducted with MDD patients receiving escitalopram or sertraline found that high baseline S100B levels correlated to a positive treatment response yet did not result in a change in baseline S100B over time [33].

Similarly to antidepressant treatment response, higher baseline levels of S100B have been associated with better response to ECT [34]. However, S100B levels did not change significantly over time or in response to treatment, regardless of remission status, suggesting that it is not a state marker of depression. Another study (*N* = 22) found no association of baseline S100B with clinical efficacy of ECT after controlling for age [35].

One study examined S100B in ketamine treatment for MDD. In a randomized controlled trial, plasma S100B levels in SSRI-resistant MDD patients treated with ketamine (*N* = 20) or placebo (*N* = 10) were not significantly different between responders and non-responders. Additionally, no significant change in S100B levels was observed following ketamine treatment [36].

Recent studies suggest that S100B levels may be modulated by TMS in MDD, although its role as a biomarker for treatment response is unclear. One study found that patients undergoing both rTMS and antidepressant treatment had significantly decreased S100B levels compared to baseline, with a second study replicating the findings of reduced S100B levels in patients undergoing rTMS [37,38]. Like other studies, baseline S100B was found to be higher in MDD patients versus healthy controls. Neither study differentiated between treatment responders and non-responders. These findings suggest that TMS may engage neurobiological mechanisms distinct from other depression treatments, potentially involving glial modulation and neuroplasticity. Further research is needed to clarify whether S100B can serve as a biomarker predictive of treatment response in TMS.

#### 3.2.2. S100B in Perinatal Depression

There is a paucity of research on S100B in perinatal or postpartum depression. We identified one study reporting that SSRI treatment was associated with increased S100B in pregnant women. However, the study did not subclassify participants by prior depression diagnosis, limiting its interpretability [25]. There is a clear gap in the literature, underscoring the need for further investigation into S100B in perinatal and postpartum populations, particularly in relation to both diagnosis and treatment response.

### 3.3. Electroencephalography (EEG)

#### 3.3.1. EEG in MDD

EEG metrics have been extensively explored as a non-invasive biomarker for predicting treatment response in MDD, although findings remain mixed. Research has explored several different EEG metrics in relation to treatment response as well as machine-learning algorithms.

Alpha activity, specifically left-sided frontal alpha asymmetry (FAA), was initially proposed to predict response to antidepressant treatment. However, one meta-analysis showed considerable heterogeneity in that FFA did not consistently predict treatment response or even differentiate depressed participants from healthy controls [39]. A subsequent meta-analysis in a low-heterogeneity selection of studies found non-significant tendency towards left lateralization in depressed patients [40]. A more recent meta-analysis showed a small but significant effect size for FAA being associated with MDD, despite considerable heterogeneity of the studies examined, suggesting limited diagnostic value [41].

While FAA may not predict treatment outcomes in depressed patients in general, there is evidence that it may do so in a subset. Notably, in the large iSPOT-D trial, women with relatively greater right-hemisphere alpha power at baseline were more likely to respond to the SSRI antidepressants, escitalopram, or sertraline [42]. This FAA predictor was specific to gender and treatment, as it was not seen in men or with venlafaxine. Overall, the evidence for FAA as a biomarker for depression and treatment response is mixed.

A wide variety of EEG metrics exist. One meta-analysis examining 47 studies identified theta band density and cordance as having the greatest promise for potential EEG biomarkers in regard to treatment response [43]. Theta band density reflects the amount of activity within the theta frequency range, localized to a region of interest. In patients with MDD, the preponderance of studies examined demonstrated elevated theta current density at baseline in the rostral anterior cingulate cortex (ACC) and the medial orbitofrontal cortex (mOFC) in treatment responders. This included response to SSRIs, SNRIs, dopamine reuptake inhibitors, norepinephrine reuptake inhibitors, and, interestingly, response to placebo [44].

Cordance is a composite measure that incorporates absolute and relative power to estimate regional brain activity relative to all brain activity in regions of interest and it may detect changes in cortical function in these specific regions. A systematic review found that decreased prefrontal theta cordance after 1 week of antidepressants is associated with positive treatment response, with mixed evidence in neuromodulation treatment [43]. Other studies in a more recent meta-analysis looked at available biomarkers that can predict treatment response in rTMS for MDD specifically, finding that frontal theta cordance was the most promising [45]. However, replication of results and further clarification of the underlying mechanism is required, with the caveat that a composite of multiple EEG metrics will likely be more meaningful.

Other EEG metrics that may be fruitful include individual alpha peak frequency (iAPF). TMS studies have demonstrated that greater clinical improvement is associated with slower iAPF in individuals [46,47], with other studies not replicating this result [48,49]. However, iAPF is likely related to treatment response in a nonlinear manner [50,51], with less absolute distance between 10 Hz and iAF to be associated with response [50,52]. IAPF scores have also been associated with antidepressant response, with slower iAPF associated with response to sertraline [53,54]. Voetterl and colleagues demonstrated iAPF association with treatment response in multiple datasets, proposing Brainmarker I as a method to stratify treatment decisions based on iAPF by assigning patients with mid to high decile to different TMS protocols and patients with lower decile to ECT [54,55]. iAPF represents a promising biomarker candidate that would benefit from further replication and validation.

One meta-analysis examined quantitative EEG measures in general, including event-related potentials, to assess accuracy for treatment response prediction. While predictive power for all biomarkers pooled together was above chance, no single biomarker showed significantly greater predictive power than others. Analysis suggested significant publication bias and small sample size [56]. While particular EEG metrics may have potential as treatment response biomarkers, further large sample studies are necessary.

Analyzing EEG metrics via machine learning is another potentially fruitful avenue. A recent meta-analysis included 15 studies examining treatment response in MDD, finding that EEG models were able to predict treatment response with >80% accuracy. Interestingly, performance was higher in identifying non-responders rather than responders. Studies examined treatment response to antidepressants, ketamine, and rTMS [57].

#### 3.3.2. EEG in Perinatal Depression

EEG studies examining depression in perinatal or postpartum depression are scarce. One study examined resting state EEG during the 38th week of pregnancy, in women with perinatal depression (*N* = 13) compared with healthy pregnant controls (*N* = 20). Results demonstrated decreased brain network connectivity in perinatal depression, as well as >80% accuracy in the classification of patients with perinatal depression from healthy controls [58]. Further work by the same group demonstrated that these differences were specific to two EEG networks, cortical space phase locking value networks and large-scale networks [59]. No research on EEG in perinatal/postpartum treatment response currently exists, to the best of our knowledge.

### 3.4. Event-Related Potentials (ERPs)

#### 3.4.1. ERPs in MDD

Event-related potentials (ERPs) have also been examined as potential markers of treatment response. ERPs are time-locked EEG responses to stimuli, such as viewing a face or hearing a noise. This signal is typically averaged across many trials to isolate the brain’s electrical response. Among the various ERP components studied in MDD, two measures have emerged as promising for predictive utility.

One well-studied ERP component is the P300, which reflects cognitive–emotional processing and attention allocation. Recent meta-analysis of 127 studies (*N* = 12,722) examining P300 amplitude or latency demonstrated a small but significant correlation for both reduced amplitude and increased latency with depression after correcting for publication bias [60]. Studies examining treatment response have reported changes in P300 measures: in adults with MDD treated with antidepressants, changes included prolonged P300 latency and reduced amplitude [61,62]; in adolescents, decreased P300 amplitude correlated with sertraline efficacy [63]; and in children, P300 changes in response to tasks were associated with psychotherapy response [64]. Further investigation into P300 changes as a state-related treatment response biomarker is warranted, given the promising results in relation to treatment response.

Another ERP-based marker with relatively well-established predictive potential is the Loudness Dependence of Auditory Evoked Potentials (LDAEP). LDAEP quantifies the slope of the amplitude increase in the N1/P2 auditory components in response to progressively louder auditory stimuli. It is believed to be inversely related to central serotonergic neurotransmission, where a steeper slope reflects lower serotonergic activity, although the evidence for this in humans is conflicting [65]. One meta-analysis found that in patients with depression and anxiety, higher baseline LDAEP predicted favorable response to SSRIs, with the effect remaining significant after correcting for publication bias [66]. Six of the seven studies in this meta-analysis focused on depression. This finding was supported in a study examining treatment response in MDD patients treated with escitalopram or duloxetine [67]. Interestingly, evidence suggests that LDAEP may be differentially associated with response to the norepinephrine reuptake inhibitor reboxetine, with low LDAEP significantly associated with treatment response [68,69]. Given these findings, LDAEP is a promising biomarker that potentially may predict response to antidepressants, with intriguing preliminary evidence for differentially predicting response to serotonin versus norepinephrine reuptake inhibitors.

#### 3.4.2. ERP in Perinatal Depression

The search for research on ERP predictors for patients with perinatal/postpartum depression yielded no results. Given the non-invasive nature of ERP, examining the potential as a biomarker in the perinatal period is especially recommended. Alterations in P300 and LDAEP have shown promise as predictors of treatment response in MDD and may represent a fruitful avenue for investigation in perinatal depression.

### 3.5. Metabolomics

#### 3.5.1. Metabolomics in MDD

Metabolomics is an emerging approach in the identification of potential biomarkers for psychiatric disorders. It involves the comprehensive analysis of small-molecule metabolites derived primarily from lipid and carbohydrate metabolism, reflecting the integrated effects of genetic, environmental, and lifestyle factors. While metabolic profiles can be obtained from various bodily fluids, peripheral blood is most commonly used for these analyses. Metabolic profiles are obtained via chromatography followed by spectroscopy. Metabolomics also has the potential of elucidating possible pathways that could help identify treatment targets and contribute to the effectiveness of treatments.

In the context of MDD, several small-scale studies have demonstrated the potential utility of metabolomics in predicting antidepressant treatment response. For instance, metabolic signatures have been associated with differential response to sertraline versus placebo [70,71], as well as citalopram and escitalopram [72,73]. Additionally, one study examined metabolomic effects in patients with treatment-refractory MDD who were treated with ketamine or esketamine, compared to placebo. No baseline metabolite signatures were found to be associated with treatment response [74].

#### 3.5.2. Metabolomics in Perinatal Depression

Several metabolites have been reported as altered in perinatal and postpartum depression, including those related to amino acids, the tryptophan–kynurenine pathway, purines, steroids, and neurotransmitters [75]. However, to our knowledge, no studies to date have specifically examined the role of metabolomic markers in predicting treatment response in perinatal/postpartum depression. Future research in this area holds the potential to enhance treatment strategies for perinatal depression and advance our understanding of its unique biological underpinnings.

### 3.6. Hypothalamic–Pituitary–Adrenal (HPA) Axis

#### 3.6.1. The HPA Axis and MDD

Dysregulation of the HPA axis has long been implicated in depression and can lead to downstream effects on other physiological systems. In response to stress and circadian rhythms, the hypothalamus releases corticotropin-releasing hormone (CRH), prompting adrenocorticotropic hormone (ACTH) to be secreted by the pituitary gland, which in turn causes the adrenal cortex to secrete cortisol. Increased cortisol and ACTH have been associated with depression [76]. One meta-analysis of depression biomarkers found that increased baseline cortisol showed a modest predictive effect on onset, relapse, and recurrence of MDD [77]. Depressed individuals are shown to have higher levels of corticotrophin-releasing hormone in cerebrospinal fluid [78].

Cortisol has been an HPA axis biomarker of particular interest. No meta-analysis specific to treatment response could be found. One review found elevated baseline cortisol levels in severe and acute MDD but found no convincing relationship between cortisol level and treatment response with antidepressant medications [79]. Of note, cortisol levels are subject to a wide array of variables, including the menstrual cycle, oral contraceptive pills, psychosocial stressors, and more. One such variable that is likely mediating this effect is age. One retrospective analysis found that cortisol, moderated by age, was significantly associated with MDD and reduction in depression symptoms. Specifically, low cortisol was associated with lower MDD diagnosis rate and higher antidepressant efficacy in participants below median age, while in participants above median age, lower cortisol was associated with high MDD diagnosis rate and less improvement in depression with treatment [80].

Other findings regarding HPA axis markers are less robust. A meta-analysis of 34 studies (*N* = 1049), found no significant changes in cortisol or ACTH levels after various depression treatments, including antidepressants, lithium, ECT, and rTMS [81]. Similarly, a meta-analysis of 39 studies investigating HPA functioning in relation to antidepressant medications revealed no significant differences in pretreatment levels of CRH or ACTH between treatment responders and non-responders. Basal cortisol was found to be higher in non-responders in studies that measured cortisol in urine or saliva rather than blood. This association was found in studies that did not adjust for participant age, suggesting some positive results may be inflated by age as a confounder [82].

Studies examining ketamine and cortisol response in MDD are scarce. In healthy controls, ketamine has been found to increase cortisol levels [83]. A recent placebo-controlled trial examined baseline HPA-axis hormones in patients receiving a single infusion of ketamine for MDD. It found that pre-infusion plasma CRH and ACTH levels did not significantly moderate or predict the antidepressant effects of ketamine [84]. Further investigation on the longitudinal effects of ketamine on the HPA axis is warranted.

A systematic review of 15 studies of non-convulsive neurostimulation treatments (rTMS, transcranial direct-current stimulation, VNS, and electroacupuncture) found that depressed patients undergoing rTMS showed reductions in HPA hyperactivity post-treatment across studies, including decreasing elevated cortisol and ACTH, both acutely and long-term. However, critically, changes in HPA-axis hormones did not consistently correlate with clinical symptom improvement in these studies [85].

#### 3.6.2. The HPA Axis and Perinatal Depression

In perinatal and postpartum depression, findings are mixed. Cortisol awakening response is the rapid increase in cortisol that occurs within an hour of waking, reflecting increased HPA axis activity. A systematic review of 47 studies examining cortisol and perinatal depression found evidence of blunted cortisol awakening response in maternal depression [86]. However, another systematic review revealed that studies have not been consistently able to replicate a meaningful correlation between cortisol and perinatal depression [87]. Given that pregnancy is associated with alterations in the HPA axis, these mixed findings are not surprising. No study to date has specifically investigated treatment response in relation to HPA axis markers in perinatal depression. Since perinatal depression may involve distinct pathophysiological mechanisms compared to MDD, given the dramatic neuroendocrine changes during pregnancy, it is plausible that HPA axis biomarkers could offer unique predictive value.

### 3.7. Neuroimaging Markers

#### 3.7.1. Neuroimaging Markers in MDD

Investigation in neuroimaging biomarkers for treatment response has identified a number of brain areas that are associated with treatment response in MDD. The bulk of studies in this field has focused on the modalities of structural and functional MRI (fMRI). A meta-analysis of 29 MRI studies identified increased baseline activity in the ACC as predictive of treatment response [88]. Additionally, increased baseline activation in the striatum and insula, and decreased right hippocampal volume were associated with lower likelihood of response. Treatment was either an antidepressant medication or cognitive behavioral therapy. Earlier meta-analysis examining 23 studies also identified increased ACC activity to be related to response to various treatments, including antidepressants, rTMS, ECT, or sleep deprivation [89].

One review examined neuroimaging biomarkers predicting response to ECT in 19 studies. Lower hippocampal volume, increased amygdala volume, increased subgenual cingulate gyrus volume, and connectivity between the dorsolateral prefrontal cortex and posterior default mode network were identified as predictive of treatment response to ECT [90]. A multimetric review of 34 studies found increases in functional connectivity in the superior, middle frontal gyri, and the subgenual ACC to be related to response to ECT [91]. Dysregulation in frontal areas have been implicated in meta-analyses as a potential state biomarker [92]. A meta-analysis focused on resting state functional connectivity in MDD patients undergoing ECT, rTMS, or psychotherapy revealed that patterns within the frontoparietal and default mode networks were associated with treatment response [93].

For ketamine, fewer studies exist and no meta-analyses were found in our search. Several biomarkers related to treatment response have been implicated in small studies, including the increased activity in the pregenual ACC [94], increased fractional anisotropy in the cingulum and forceps minor [95], increased global signal regression values in the lateral prefrontal cortex, caudate, and insula [96], decreased default mode network deactivation and decreased dorsolateral prefrontal cortex activation [97], and decreased connectivity between cerebellum and frontoparietal and sensory motor networks [98]. Additionally, increased hippocampal gray matter volume was associated with treatment response to oral ketamine in MDD patients with suicidality [99].

#### 3.7.2. Neuroimaging Markers in Perinatal Depression

To our knowledge, no studies exist that examine neuroimaging biomarkers predicting treatment response in perinatal depression. While individual studies have examined general neuroimaging biomarkers in perinatal depression, finding alterations in the hippocampus, ACC, amygdala, medial prefrontal cortex, and striatum [100], no meta-analyses exist.

### 3.8. Inflammatory Markers

#### 3.8.1. Inflammatory Markers in MDD

A large number of peripheral inflammatory biomarkers have been studied. IL-8, a signaling molecule that activates neutrophils, is of leading interest. Specifically, IL-8 is significantly lower at baseline in antidepressant responders compared to nonresponders in meta-analyses [101,102].

C-reactive protein (CRP) has also been studied. Recent meta-analysis demonstrated that lower baseline CRP was associated with response to antidepressant therapy [102], with non-significant association with lower levels in earlier meta-analysis [101].

In earlier meta-analysis, TNF-α levels were not decreased in patients receiving antidepressant treatment; however, subgroup analysis demonstrated significant difference by medication class, with trending decrease in studies examining SSRI treatment [103]. Subsequent meta-analysis found significant decrease in TNF-α after antidepressant treatment in responders compared to controls and non-responders [101], as well as in a meta-analysis specific to SSRIs [104].

Other examined cytokines have produced notably mixed data. IL-4, IL-6, IL-10, and IL-1β have had no significant treatment effects observed in analysis by treatment response [101]; however, demonstrated significance in analyses is not specific to response. One meta-analyses found significant decreases in IL-4 after antidepressant treatment [105], although this was not replicated in a meta-analysis specific to SSRIs [104]. IL-6 was found to be significantly decreased after antidepressant treatment [103,105] and SSRIs [104]. IL-10 was significantly decreased after antidepressant treatment [105] and SSRIs [104]. IL-1β was found to be reduced after antidepressant treatment [103] and after SSRIs [104,105]. IL-5 and granulocyte–macrophage colony-stimulating factor (GM-CSF) levels were also significantly decreased in responders after antidepressant treatment [101]; however, this result was underpowered.

Evidence for inflammatory biomarkers in the context of neuromodulation and ketamine treatments is comparatively sparse. A meta-analysis demonstrated that higher baseline CRP and IL-6 levels were associated with ECT response, with correlation in IL-8 and kynurenine metabolites [106]. There are a few studies on treatment response to ketamine. A meta-analysis of inflammatory markers showed no significant changes, longitudinally, in pro-inflammatory markers in ketamine/esketamine responders or non-responders [107]. A search for results via TMS yielded one study, a double-blinded RCT examining theta burst stimulation in MDD patients, finding no correlation in serum inflammatory cytokines and depressive symptoms [108].

#### 3.8.2. Inflammatory Markers in Perinatal Depression

Pregnancy represents a period of altered immune and inflammatory function. In general, studies on inflammatory markers during the antenatal and postpartum periods have linked depression symptoms to various inflammatory markers; however, no conclusions can be drawn due to the wide heterogeneity amongst studies [109]. However, while examination of inflammatory markers has been comprehensive in MDD, no meta-analyses specific to treatment response in perinatal depression were found in our search. One cross-sectional study (*N* = 723) examining treatment responders was identified. Depressive symptoms in pregnant patients between 12 and 21 weeks were examined, finding that responders to pharmacotherapy had significantly lower concentrations of TNF-alpha compared to non-responders and untreated participants [110]. No significant differences were found for IFN-γ, IL13, IL-6, IL-8, and CRP. Additionally, one study examined inflammatory markers in pregnant women, finding that women who took SSRIs had lower levels of IL1β, IL-5, and IL-10 [111]. However, this study did not examine treatment response and primarily compared groups on receiving SSRIs rather than perinatal depression diagnosis.

Given the unique immune adaptations of pregnancy, investigating inflammatory markers in perinatal depression may identify biomarkers that guide personalized treatment strategies. Findings from MDD point to IL-8, CRP, and TNF-α as candidate biomarkers, and one antenatal study [110] linked lower TNF-α concentrations with better treatment response. However, evidence remains sparse and treatment-focused studies are needed to determine whether inflammatory markers can be translated into clinically useful tools for optimizing care in perinatal depression.

### 3.9. Neuroactive Steroids

#### 3.9.1. Allopregnanolone (ALLO) and Perinatal Depression

Neuroactive steroids are endogenous steroid molecules that rapidly modulate neurotransmitter receptors. They play a complex role in the pathophysiology of perinatal depression. The HPA axis, mediating stress responses in the human body, is closely regulated by GABAergic signaling provided by neuroactive steroids. Key neuroactive steroids implicated in mood disorders include allopregnanolone (ALLO) as well as dehydroepiandrosterone (DHEA) and its sulfate (DHEA-S). ALLO and Progesterone (P4) are isomers, both positive allosteric modulators of the GABA_A_ receptor. It has been demonstrated that women with a history of postpartum depression are sensitive to induced hypogonadism and, under these conditions, are more likely to develop depressive symptoms compared to women without a history of PPD [112].

The preponderance of studies on neuroactive steroids is on ALLO. During pregnancy, ALLO is universally increased, with a drop in levels after delivery. Understanding the exact relationship of ALLO levels to perinatal depression has been challenging due to conflicting results. In one meta-analysis, ALLO concentrations during pregnancy did not differ when comparing women who had peripartum depressive symptoms and those who did not. In a subgroup analysis, higher ALLO was detected in women with depressive symptoms at gestational weeks 21–24 and 25–28 when examining patients not receiving antidepressants [113]. Delineating the exact relationship of ALLO to perinatal depression remains challenging due to fluctuations throughout pregnancy and differing of assays used to detect ALLO. No analyses, to our knowledge, have been conducted on ALLO as a biomarker for treatment response.

However, the efficacy of brexanolone and zuranolone (an oral synthetic analog of ALLO), both FDA-approved for postpartum depression, provide clear evidence for the antidepressant role of ALLO in perinatal depression. Brexanolone, an exogenous form of intravenous ALLO, was approved by the FDA in 2019 for postpartum depression after demonstration that it was effective in rapidly treating postpartum depression in clinical trials [114,115]. Additionally, the dynamic changes in neuroactive steroid levels and ratios of pregnanolone metabolites compared to neuroactive steroids over the course of pregnancy may affect perinatal depression. Surges in ALLO and P4 have been demonstrated to predict increases in perinatal depression during the first and second trimesters [116]. Although the direction of the association between ALLO levels and perinatal depressive symptoms has not been conclusively established, treatment efficacy has consistently demonstrated a clear relationship.

It has been noted in rodent models that changes in subunit expression of the GABA_A_R can lead to depressive behaviors [117,118,119]. Additionally, subunit changes have been associated with the fluctuation of progesterone and estradiol during pregnancy [120,121,122]. Further investigation in humans on the association of receptor subunits and delineation of the dynamic changes during pregnancy and in association with PPD is needed to clarify the exact relationship of ALLO to PPD. Such studies may elucidate novel pathophysiological mechanisms and clarify the potential utility of ALLO as a biomarker.

#### 3.9.2. ALLO in MDD

As for MDD, two studies in 1998 demonstrated that ALLO in plasma and CSF was decreased in MDD compared to controls, with ALLO levels increasing after SSRI treatment [123,124]. Additionally, the study examining ALLO in CSF demonstrated that larger increases in ALLO were associated with greater improvement in depressive symptoms, and that ALLO was only significantly increased in responders [123]. One study demonstrated that females with MDD in remission had higher levels of progesterone and ALLO than controls [125]. One study investigated neuroactive steroids in MDD patients undergoing rTMS monotherapy for 8 weeks. Patients had significantly lower ALLO levels after undergoing rTMS [126]. No specific analysis was conducted for responders. Trials have also been conducted for treatment with zuranolone in MDD. Phase three randomized clinical trials demonstrated mixed results, and it has yet to be FDA-approved for MDD outside of the perinatal period [127,128].

#### 3.9.3. DHEA and DHEA-S in MDD

DHEA and DHEA-S, synthesized in the adrenal cortex, have broad effects throughout the brain on various neurotransmitters and receptors. DHEA-S circulates at higher concentrations, has a longer half-life, and is biologically more inactive than DHEA. It can be converted back to DHEA in tissues. Two meta-analyses examining DHEA-S expression showed lower expression in depressed patients; however, this was not true in subgroup analyses of Caucasian and Asian patients [129,130]. A meta-analysis of two studies on DHEA and four on DHEA-S did not demonstrate any differences in women who were depressed versus controls, although it noted that studies examining DHEA-S in plasma found lower levels in depressed women [131]. One more recent study examining both DHEA and DHEA-S in saliva found that DHEA levels, but not DHEA-S, were lower in patients with recurrent MDD compared to patients with first-episode MDD [132].

Several studies have examined DHEA and DHEA-S, in relation to treatment response, with mixed results. One study in elderly patients found no difference in baseline DHEA/DHEA-S levels but did find lower DHEA/DHEA-S in remitters compared to non-remitters after treatment with nortriptyline or paroxetine [133]. In contrast, venlafaxine-treated patients showed no difference in DHEA between remitters and non-remitters [134]. Other studies reported higher baseline DHEA and DHEA-S in SSRI remitters compared to non-remitters and controls [135], and higher baseline DHEA in responders across a range of psychotropics in an inpatient setting [136]. No significant difference was found in DHEA levels in remitters compared to non-remitters in another study [124]. More recently, patients receiving rTMS monotherapy for MDD demonstrated significantly lower DHEA and DHEA-S compared to healthy controls [126].

Similarly to other neuroactive steroids, attention has been turned towards supplementation as treatment. In a meta-analysis, DHEA supplementation was found to be associated with decreased depressive symptoms compared to placebo; however, evidence was considered low-grade [137]. Taken together, DHEA/DHEA-S is tentatively associated with lower levels in patients with MDD; however, the directionality of levels for treatment response has been conflicting, likely due to differences in demographic variables and antidepressant treatments. Given the heterogeneity of study results, DHEA/DHEA-S as a biomarker for treatment response with antidepressants requires further study.

#### 3.9.4. DHEA and DHEA-S in Perinatal Depression

Levels of DHEA/DHEA-S throughout pregnancy have been difficult to characterize, with conflicting results from a variety of studies. A large study of mothers (*N* = 1983) found lower DHEA in patients with postpartum depression [138]. One study on pregnant patients (*N* = 40) found a trend towards increased DHEA-S in patients with perinatal depressive symptoms; however, this was not significant [139]. Another study examining DHEA in hair samples found that higher prenatal levels of DHEA and lower postnatal levels of DHEA were able to predict whether participants had PPD with 98% accuracy [140]. No studies were found examining treatment response in perinatal depression. Similarly to ALLO, DHEA levels are dynamic throughout pregnancy. Prior to consideration as a potential biomarker, further delineation of baseline levels and alterations in perinatal depression would clarify the potential of DHEA/DHEA-S as a biomarker.

## 4. Discussion

The identification of biomarkers predictive of treatment response in MDD has advanced considerably over the past two decades, with particular focus on neurotrophic factors, neuroendocrine markers, inflammatory mediators, and neuroimaging-based signatures. Although perinatal depression has historically been underrepresented in biomarker research, emerging evidence suggests that certain markers, such as BDNF, neuroactive steroids, neuroimaging findings, and inflammatory markers, may hold translational relevance in this unique population.

BDNF, with strong evidence for association with antidepressant response in MDD [15,17], is of particular interest for further investigation in perinatal contexts. Recent findings that BDNF may mediate response to dexmedetomidine in postpartum depression reinforce its potential utility as a biomarker [26]. Elevated baseline S100B levels have been consistently associated with treatment response in MDD [30,31,32]. However, no studies examining S100B in relation to treatment response in perinatal depression were found, representing an opportunity for investigation.

Of EEG metrics, theta cordance is the most promising given that decreased activity is significantly associated with positive treatment response [43,44,45]. No studies on EEG metrics for treatment response in perinatal depression were found. Higher baseline LDAEP has also predicted favorable response to SSRIs and SNRIs [66,67], but no studies exist on ERP biomarkers in perinatal depression.

Metabolomics, an emerging field, the association of metabolomic signatures with response to medications in MDD [70,71,72,73]. Several metabolites have been shown to be altered in perinatal depression, including alterations in metabolism of tryptophan, amino acid, steroid, neurotransmitters, and steroids [75], representing a fruitful avenue to investigate treatment response.

Studies examining cortisol, ACTH, and CRH failed to find convincing evidence for treatment response in MDD [79,81,82,85] and no convincing relationship was found in studies examining cortisol and perinatal depression as well [87]. This is likely due to the considerable variation in methodology, collection processes, and assays. Standardization will be essential in future work to determine whether HPA-axis hormones can serve as reliable biomarkers of treatment response.

Neuroimaging studies have most consistently identified increased activity in the ACC as predictive of treatment response in MDD [88,89,91,94]. While alterations in neuroimaging biomarkers, including the ACC, have been identified in perinatal depression [100], no studies have examined treatment response. Investigating the ACC as a potential predictor of treatment response in perinatal depression therefore represents an important next step.

Inflammatory markers most consistently demonstrate lower levels of IL-8 [101,102], CRP [102], and TNF-α [101,104] in antidepressant treatment responders. One cross-sectional study found that pregnant patients responding to pharmacotherapy had significantly lower concentrations of TNF-α compared to non-responders and untreated participants [110]. Further investigation of IL-8 and CRP in relation to treatment response, along with replication of TNF-α findings, is warranted to advance perinatal depression research.

DHEA and DHEA-S was shown in meta-analyses to be associated with lower expression in MDD patients with possible interaction for race [129,130]. Studies in treatment response have demonstrated conflicting results. DHEA supplementation was associated with fewer depressive symptoms; however, this evidence was considered low-grade [137]. Levels of DHEA/DHEA-S throughout pregnancy have been difficult to characterize as well due to conflicting results, and no studies examined treatment response in perinatal depression. Further characterization of DHEA/DHEA-S would clarify its potential biomarker status.

While several MDD biomarkers demonstrated a strong level of evidence for treatment response demonstrated by meta-analyses [see Table 1], an important caveat is that this group-level difference does not necessarily translate to clinical significance or practical implementation. Indeed, no MDD biomarker has reached routine clinical use yet. Additionally, across several domains, findings are inconsistent due to methodological heterogeneity, small sample sizes, and confounding variables such as age, comorbidities, hormonal fluctuations, and medication exposure. Practical barriers such as cost, accessibility, and methodological standardization have also prevented implementation of biomarkers. BDNF, inflammatory markers, neuroactive steroids, and electrophysiological measures show promise, but require further validation. Several factors may play a role in the lack of biomarkers in depressive disorders and psychiatric disorders at large: subjective symptoms, self-report measures, and a focus on diagnostic categories rather than dimensionally varying symptoms. While some scholars may suggest abandoning biomarkers altogether, this approach risks limiting efforts to understand neurobiological mechanisms and reinforces the same barriers that have hindered progress. Research in biomarkers in less heterogeneous subtypes of depression, such as perinatal depression, may be more fruitful.

In this respect, perinatal depression has an advantage despite a comparatively smaller literature base, due to less heterogeneous pathophysiology and clearer mechanisms. Uniquely, perinatal depression has been shown to be able to be manipulated by inducing hypogonadism [112] or increasing ALLO. The efficacy of zuranolone and brexanolone as rapidly acting antidepressants for perinatal depression provide evidence for altered ALLO signaling, despite challenges in delineating an exact relationship between peripheral ALLO levels and depressive symptoms. Meanwhile, trials for zuranolone in MDD have mixed results [127,128]. Animal studies have demonstrated the fluctuation of subunit expressions within the GABA_A_ receptor associated with pregnancy [120,121,122] and further investigation of this mechanism may clarify the physiological process through which ALLO exerts an effect in perinatal depression, as well as potentially emerges as a candidate biomarker.

Conversely, perinatal depression research also offers additional challenges. Although perinatal depression shares clinical features with non-perinatal MDD, it occurs within a uniquely dynamic biological context. Pregnancy, postpartum, and breastfeeding represent periods of altered immune physiology with rapid dynamic shifts, potentially altering inflammatory markers that may signal depression states. Careful consideration should be paid to timing. For example, gestational age may impact findings, especially in neuroactive steroids, including those discussed in ALLO [113].

One limitation is the ambiguity in how individual studies diagnose patients with perinatal depression. For example, some patients may have depression that emerges or worsens during pregnancy, others may have pre-existing depressive symptoms that persist, and others may have severe MDD with symptoms that temporarily improve during pregnancy but still meet criteria. Prior depression and changes from baseline may not be carefully considered. Studies do not clearly distinguish these trajectories, even though this may impact the subsequent findings, given the possible different neurobiological mechanisms. Further analyzing the individual trajectories of depressive symptoms during the perinatal period may also improve the ability to detect meaningful signals in data.

The success of ALLO analogs underscores how biomarker-informed therapeutics can reshape clinical care and offer new insights into neurobiological mechanisms, yet also reveal that there is much that is still unknown on the basic relationship of ALLO with depression. Focus on a translation approach from the comparatively more vast MDD literature may yield new insights in perinatal depression research. Advancing promising biomarkers within the relatively less heterogeneous context of perinatal depression may provide a critical step towards achieving clinically meaningful tools.

## Figures and Tables

**Table 1:** Summary Table on Biomarkers for Treatment Response in MDD and Perinatal Depression.

Domain	Biomarker	Direction of Change in MDD Responders	Strength of Evidence	Evidence in Perinatal Depression
Neurotrophic Factors	BDNF	Increased levels after treatment	Strong in antidepressants. Limited in neuromodulation and ketamine	No studies on treatment response. Two studies linked treatment to increased BDNF.
	S100B	Higher baseline levels	Strong in antidepressants. Limited evidence against in ketamine.	No studies on treatment response. A study linked SSRI use to increased S100B.
EEG	FAA	Left-sided	Limited in antidepressants	No studies found.
	Theta band density	Elevated baseline levels in rostral ACC and mOFC	Strong in antidepressants	No studies found.
	Theta cordance	Decreased after treatment	Strong in antidepressants Moderate in TMS	No studies found.
	iAPF	Mid to fast frequency for TMS, slow frequency for sertraline and ECT	Moderate in TMS, sertraline, and ECT	No studies found.
ERP	P300	Reduced amplitude and increased latency	Moderate in antidepressants	No studies found.
	LDAEP	Higher baseline	Moderate in SSRIs	No studies found.
Metabolomics		Altered metabolic signatures	Preliminary for SSRIs	No studies on treatment response. Altered metabolites in postpartum depression.
HPA Axis	Cortisol	No consistent relationship	Strong evidence against in antidepressants, ketamine, or neuromodulation	No studies on treatment response. Inconsistent data on cortisol association.
	ACTH	No consistent relationship	Strong evidence against in antidepressants, ketamine, or neuromodulation	No studies found.
Neuroimaging	MRI	Strongest association for increased baseline activity in ACC	Strong in antidepressants and neuromodulation.	No studies found in treatment response. Implicated alterations in the ACC and other areas.
Inflammatory Markers	CRP	Decreased baseline levels	Strong in antidepressants and ECT	Limited evidence against
	TNF-α	Decreased levels after treatment	Strong in antidepressants	Limited evidence for decreased levels in responders
	IL-8	Decreased baseline IL-8	Strong in antidepressants	Limited evidence against
	IL-6	Decreased levels after treatment	Limited in antidepressants Strong evidence for ECT	Limited evidence against
	IL-4	Decreased levels after treatment	Limited in antidepressants	No studies found.
	IL-10	Decreased levels after treatment	Limited in antidepressants	No studies found. A study linked SSRI use is to lower IL-10.
	IL-1β	Decreased levels after treatment	Limited in antidepressants	No studies found. A study linked SSRI use to lower IL-1β.
Neuroactive steroids	ALLO	Increased levels after treatment with antidepressants	Moderate in antidepressants. However, zuranolone trials with mixed results.	Strong evidence for decreases in ALLO signaling.
	DHEA/ DHEA-S	Decreased levels after treatment	Limited in antidepressants	No studies found in treatment response. Mixed on association with perinatal depression.

Evidence was graded according to the following: Strong = meta-analysis level data or multiple consistent high-quality studies. Moderate = multiple consistent studies. Limited = inconsistent data or small studies. Preliminary = Few studies.

## Data Availability

No new data were created or analyzed in this study. Data sharing is not applicable to this article.

## Abbreviations

The following abbreviations are used in this manuscript:

MDD	Major depressive disorder
TMS	Transcranial magnetic stimulation
rTMS	Repetitive transcranial magnetic stimulation
ECT	Electroconvulsive therapy
BDNF	Brain-derived neurotrophic factor
VNS	Vagal nerve stimulation
SSRI	Selective serotonin reuptake inhibitor
HAM-D	Hamilton Depression Rating Scale
S100B	S100 calcium-binding protein B
FFA	Frontal alpha asymmetry
EEG	Electroencephalography
SNRI	Serotonin Norepinephrine Reuptake Inhibitor
ERP	Event-related Potentials
LDAEP	Loudness Dependence of Auditory Evoked Potentials
HPA	Hypothalamic–pituitary–adrenal
CRH	Corticotropin-releasing hormone
ACTH	Adrenocorticotropic hormone
fMRI	Functional magnetic resonance imaging
ACC	Anterior cingulate cortex
mOFC	Medial orbitofrontal cortex
